# Association of TERT, OGG1, and CHRNA5 Polymorphisms and the Predisposition to Lung Cancer in Eastern Algeria

**DOI:** 10.1155/2020/7649038

**Published:** 2020-03-20

**Authors:** Asma Mimouni, Etienne Rouleau, Patrick Saulnier, Amina Marouani, Med Lamine Abdelali, Taha Filali, Leila Beddar, Abdelhak Lakehal, Ahmed Hireche, Asma Boudersa, Mahmoud Aissaoui, Hacene Ramtani, Khalid Bouhedjar, Djamel Abdellouche, Messaouda Oudjehih, Ibtissem Boudokhane, Noureddine Abadi, Dalila Satta

**Affiliations:** ^1^Laboratoire de Biologie Moléculaire et Cellulaire, University Frères Mentouri Constantine 1, Constantine 25000, Algeria; ^2^Laboratoire de Biologie et Génétique Moléculaire, Univ. Constantine 3, Constantine 25000, Algeria; ^3^AMMICa/US 23 INSERM / UMS 3655 CNRS, Villejuif Cedex 94805, France; ^4^Service de Pneumologie, CHU Sétif, 19000, Algeria; ^5^Physics Department, Univ. Frères Mentouri Constantine 1, Constantine 25000, Algeria; ^6^Service d'Oncologie CHU Constantine, Faculté de Médecine Univ. Constantine 3, Constantine 25000, Algeria; ^7^Service d'Anatomie Pathologique, CHU Constantine, Faculté de Médecine Univ. Constantine 3, Constantine 25000, Algeria; ^8^Registre de Cancer Constantine et Service d'Épidémiologie et Médecine Préventive, CHU Constantine, 25000, Algeria; ^9^Service d'Oncologie, CHU, Annaba 23000, Algeria; ^10^Clinique Alfarabi, Annaba 23000, Algeria; ^11^Centre de Recherche en Biotechnologie, Constantine 25000, Algeria; ^12^Service D'Anatomie Pathologique, CHU Sétif, Sétif 19000, Algeria; ^13^Registre de Cancer Batna et Service d'Épidémiologie et Médecine Préventive, CHU Batna, Batna 5000, Algeria

## Abstract

Lung cancer remains the most common cancer in the world. The genetic polymorphisms (rs2853669 in TERT, rs1052133 in OGG1, and rs16969968 in CHRNA5 genes) were shown to be strongly associated with the risk of lung cancer. Our study's aim is to elucidate whether these polymorphisms predispose Eastern Algerian population to non-small-cell lung cancer (NSCLC). To date, no study has considered this association in the Algerian population. This study included 211 healthy individuals and 144 NSCLC cases. Genotyping was performed using TaqMan probes and Sanger sequencing, and the data were analyzed using multivariate logistic regression adjusted for covariates. The minor allele frequencies (MAFs) of TERT rs2853669, CHRNA5 rs16969968, and OGG1 rs1052133 polymorphisms in controls were C: 20%, A: 31%, and G: 29%, respectively. Of the three polymorphisms, none shows a significant association, but stratified analysis rs16969968 showed that persons carrying the AA genotype are significantly associated with adenocarcinoma risk (pAdj = 0.03, ORAdj = 2.55). Smokers with an AA allele have a larger risk of lung cancer than smokers with GG or GA genotype (pAdj = 0.03, ORAdj = 3.91), which is not the case of nonsmokers. Our study suggests that CHRNA5 rs16969968 polymorphism is associated with a significant increase of lung adenocarcinoma risk and with a nicotinic addiction.

## 1. Introduction

Lung cancer (LC) remains the most common cancer in the world, both in terms of new cases (2 million new cases, 11.6% of all cancers) and deaths each year (1.7 million deaths 18.4%) [1]. As for Algeria, out of a population of some 42 million, 4000 cases of new LC appear every year; it is the first cause of mortality from cancer in men and the seventh in women [1, 2]. Although cigarette consumption accounts for the largest part of this alarming figure, over 80% of LC deaths are tobacco related but only a small fraction of smokers (usually <20%) develops this disease [3, 4]. In addition, a fraction of lifelong nonsmokers will die from LC, suggesting that LC is a multifactorial disease that results from complex interactions with many genetic and environmental factors [5]. Thus, it is of great importance to perform molecular biology studies in order to gain some understanding of susceptibility factors in action in the LC appearance and identify causal variants, which can be useful in the screening, the early diagnosis, and the therapy of LC. Independent Genome-Wide Association Studies (GWAS), which are considered a gold standard for reporting genotype-phenotype associations, and several other studies have been performed with the aim of looking for single-nucleotide polymorphism (SNP) of predisposition to LC [6–8]. Among the important genes which could modify the risk of LC are TERT, CHRNA5, and OGG1, and these are the genes we have been focusing on in this study.

TERT, encoded by the TERT gene, is one of the major components of telomerase; it is a reverse transcriptase that can add a nucleotide sequence (5′-TTAGGG) to telomere end [9]. The promoter region of TERT, located at positions c.124:C>T and c.146:C>T, is considered a regulatory element of telomerase activity [10]. The rs2853669 variant, located at -245 kb upstream (Ets2 binding site) of the TERT gene, prevents Ets/TCF binding, and it has been associated with lower TERT expression and decreased telomerase activity [11]. A growing number of epidemiological studies have been conducted to determine the associations between this polymorphism and cancer risk, particularly for breast cancer and LC; however, the results were inconclusive [12].

The OGG1 gene is located at chromosome 3p26.2. Its protein is a DNA repair enzyme, having both DNA glycosylase and AP lyse (apurinic or apyrimidinic site lyase) activities to remove 8-hydroxyguanine (8-oxoG) lesion produced by reactive oxygen species [13]. The rs1052133 polymorphisms have been identified in the OGG1 gene; it is a substitution at codon 326 (Ser326Cys), located in the promoter region [14]. The Cys326 protein has been shown to have about 7 times weaker 8-hydrox-yguanine-repair capacity than Ser326 protein in a complementation assay using *Escherichia coli* mutant strain deficient in 8-oxoG [15]. It has been suggested that rs1052133 was associated with LC risk; several studies investigated this association [16–18].

CHRNA genes code for proteins that form receptors which bind to nicotine and its metabolites [19]. In a large GWAS study, Hung et al. found a strong association of LC risk and CHRNA5 rs16969968 SNP; it results in the substitution of arginine for aspartic acid at the highly conserved codon 398 (D398N) of the CHRNA5 protein [7]. In vitro studies indicate that rs16969968 decreases CHRNA5 function and favors nicotinic addiction [20]. Numerous studies have been performed to find out whether this association is direct, or whether the rs16969968 SNP is simply a proxy for increased exposure to tobacco carcinogens, which were inconclusive [7, 21–26].

In our study, we were interested in SNPs of three different genes, which were selected based upon GWAS and meta-analyses of multiple populations TERT (promoter region rs2853669, c.-124C>T, and c.-146C>T), OGG1 (rs1052133), and CHRNA5 (rs16969968). The aim of our study is to investigate how these SNPs can modify the risk of LC in the Eastern Algerian population. It is important to mention that our population, to the best of our knowledge, has not been the object of any molecular study concerning the LC till now, and thus this study, as any other pioneering work, had to deal with a relative scarcity of data.

Although this study has been conducted within various population centers in Eastern Algeria which thus encompass several ethnic groups, Arabs and Central Algeria Berbers, yet the population mixing which took place at an accelerated pace after Algeria's independence, the standardization of lifestyles, the rural exodus, all concurred to homogenize the genetic stock to a large extent [27]. Furthermore, the complex processes of admixture and isolation at work has made the genetic heterogeneity found in Algeria not strongly correlated with geography or culture. It would thus be improper to assume that the various population groups to be genetically isolated and immune of gene flow [27, 28].

## 2. Materials and Methods

### 2.1. Study Population

We have performed in this work a case control study of 144 LC patients and 211 healthy controls. The patients were first diagnosed with NSCLC between June 2015 and July 2016. They came from a wide zone spanning five Wilayas (territorial units) in Eastern Algeria between governmental University Hospitals (Constantine, Sétif, Batna, and Annaba) and private clinics. There was no restriction on gender, histologic subtype, or stage with the exception of patients who refused to participate in the study. Diagnosis was made by cytological imaging and histopathological examinations. We feel confident as explained previously that our samples are largely homogeneous. In addition to that, the democratization of access to medical treatment, freely provided to all, as shown in the fact that city dwellers and those from rural areas are equally represented in the various patient samples, allowed us to make the claim of genetic homogeneity of the samples with a good degree of confidence.

The control group comprised individuals with no history of cancer and without lung-related disease to avoid possible interference from overlapping genes. They were chosen from a pool of volunteers in the blood transfusion centers during the same period of patients without restriction of age or gender. All subjects were Algerians residing in Eastern Algeria.

We used a standard epidemiological questionnaire submitted to patients and controls, and we used the medical files to collect personal data, including residential region, age, gender, profession, smoking status, symptoms, family history of cancer, histologic subtype, and stage TNM.

### 2.2. Ethics Statement

Our research has been approved by the local Ethics Committee. The use of human blood sample and the protocol in this study strictly conformed to the principles expressed in the Declaration of Helsinki, and informed consent was obtained from all participants or from their family members.

### 2.3. DNA Extraction and Genotyping

Genomic DNA was extracted from six to eight milliliters of peripheral venous bloods, which was collected into a tube containing ethylene-diamine-tetraacetic acid (EDTA) from each participant. We used standard protocols following the salt extraction procedure. The DNA concentration was measured with NanoDrop One spectrophotometer. For genotyping rs16969968 and rs1052133, we used Polymerase Chain Reaction (PCR) TaqMan; with ViiA 7 (Applied Biosystems) and TaqMan® SNP Genotyping assay kits (Applied Biosystems), we used the standard protocol recommended by the manufacturer.

The promoter TERT was amplified by PCR, and the PCR primers used were as follows: 5′ACCGTTCAGTTA GCGATTTCCCACGTGCGCAGAGGAC3′ (forward), and R: 5′CGGATAGCAAGCTCGTCTCCCAGTGGA TTCGCGGGC3′ (reverse). Then, it was genotyped with Sanger sequencing in both the forward and reverse directions using the Big Dye Terminator Chemistry V.I and an automated fluorescent sequencer, an ABI 48 Capillary Array Sequencer (Applied Biosystems 3730). Data management and analysis were done using SeqScape. For TERT sequencing, we could do sequencing for only 126 cases and 94 controls.

### 2.4. Statistical Analysis

To estimate sample size, we used functions from the R package “epiCalc” (Version 2.9.0.1) with a minor allele frequency data obtained from NCBI (http://www.ncbi.nlm.nih.gov/SNP/) and a study power fixed at 80%. The Hardy–Weinberg equilibrium of the genotype distributions is evaluated in the control subjects using a function from R package “Hardy–Weinberg” (Version 1.6.1).

The strength of association between the three polymorphisms and LC risk is assessed by calculating odds ratios (ORs) with the corresponding confidence intervals (95% CI). The ORs are also performed for a heterozygote model (AA vs. aa, where A: major allele and a: minor allele), homozygote model (Aa vs. AA), recessive model (AA+Aa vs. aa), and dominant model (AA vs. Aa+aa). We used Pearson's chi-square test to compare distributions of demographic variables, smoking status, and genotypes of the three genes between cases and controls. To calculate ORs and 95% CI, unconditional logistic regression analyses are performed. In addition, multiple unconditional logistic regression analyses with adjustment for possible confounders (age, sex, and smoking) are performed to calculate adjusted ORs and 95% CI. Statistical tests along with ORs and corresponding CIs are done using the publicly available packages of R Project for Statistical Computing (R version 3.5.1) and especially functions from packages MASS and questionr (Version 0.7.0). All statistical analyses are two sided, and the significance level is set at *p* = 0.05.

## 3. Results

### 3.1. Subjects Characteristics

To test the association between TERT, OGG1, and CHRNA5 gene polymorphisms and the risk of LC, we conducted a case-control study consisting of 144 NSCLC cases and 211 controls enrolled in four University Hospitals of Eastern Algeria (Constantine, Sétif, Batna, and Annaba). The distributions of age, gender, smoking history, and histology type among the study subjects were summarized in Table 1. The distribution of gender and that of age were statistically significant between the cases and controls (*p* < 0.01). Thus, the cases and controls were incompletely matched in this study population. Among the cases, 66% were diagnosed with adenocarcinoma and 34% with squamous cell carcinoma. Approximately 73% of case subjects and 37% of control subjects were ever smokers. As expected, case subjects had a significantly higher level (*p* = <0.01) of smoking than control subjects.

### 3.2. Association Analysis of Candidate SNPs with LC Risk

The distribution of TERT, OGG1, and CHRNA5 gene polymorphisms in cases and controls are presented in Table 2. The allele frequencies of the three gene polymorphisms in controls were consistent with the Hardy–Weinberg equilibrium; the *p* values are as follows: 0.15 for TERT, 0.49 for OGG1, and 0.15 for CHRNA5.

After sequencing the promoter of TERT gene, the two SNPs could not be found in this population (c.124C>T, c.146C>T), but we observed the presence of the rs2853669 polymorphism with a high frequency.

The minor allele frequencies (MAFs) of TERT rs2853669, OGG1 rs1052133, and CHRNA5 rs16969968 and polymorphisms in cases/controls were C: 24%/20%, G: 26.4%/29%, and A: 35.7%/31%, respectively. The genotype distribution of TERT and OGG1 was not significantly different between LC and control groups in all models, although the genotype distribution of CHRNA5 was significantly different in the recessive and homozygote modes (*p* = 0.02 and *p* = 0.03, respectively). After adjustment for the sex and age, the signification becomes marginal (data not shown), and after adjustment for the sex, age, and tobacco consumption factors, we found that the correlation vanished (pAdj = 0.16, OR = 1.83, CI = (0.78 − 4.48), and pAdj = 0.25, OR = 1.75, CI = (0.68–4.69), respectively).

### 3.3. Stratified Analysis for the Three SNPs Studied with LC Risk

We then performed a stratified analysis according to, tobacco consumption, histological type (Table 3), age, and sex (Table 4), in order to investigate associations between genetic polymorphisms and stratified factors in dominant, codominant recessive models. In addition to that, we studied these associations at the level of alleles (Table 5).

For TERT rs2853669, the results showed that the persons having CT or CC genotypes and passed 60 years had a larger risk of suffering from LC than those carrying TT genotype (*p* = 0.04). Among males, the homozygote genotype CC increase the risk of LC than TT or TC genotypes with *p* = 0.03 (Table 3). In addition to that, the patients with CC genotype have a tendency to have a squamous type rather than adenocarcinoma. Yet after adjustment, there was no statistical signification for the three stratified factors.

The OGG1 rs1052133 did not show any signification with stratified factors, except for age (>60 years), which reveals an association with the increase of LC for the persons having a minor allele (GG), in comparison with person with CC genotype (*p* = 0.04). After adjustment for age, sex, and smoking status, the signification become marginally significant (*p* = 0.09, OR = 0.49, CI = (0.21-1.11)).

As for CHRNA5 rs16969968, we observed an increasing risk for LC among males with a recessive genotype (AA) compared with those having GG or GA genotype with *p* = 0.02. However, after adjustment, the signification disappears. Smokers with an AA allele have a larger risk of LC than smokers with GG or GA genotype (*p* = 0.02); after adjustment for age and sex, the signification persists with pAdj = 0.03, ORAdj = 3.91, and CI = (1.24 − 17.34). For the stratified analysis according to the histological type, we observed that the persons with AA allele have preferentially an adenocarcinoma rather than squamous type under the recessive and homozygote models *p* = 0.003 and *p* = 0.005, respectively. This risk was not affected by adjusting for other factors (age, sex, and smoking) pAdj = 0.03, ORAdj = 2.55, and CI = (1.07 − 6.35).

As for the stratified analysis by sex, smoking, histological type, and age, according to alleles, no significant association was found.

## 4. Discussion

High incidence and poor prognosis of LC make it a major health problem worldwide. Although the LC is linked to environmental exposure to carcinogens, especially cigarette smoking, only some smokers develop LC, which suggests that there is an interindividual difference in susceptibility to the disease. In this study, polymorphisms in three genes involved in the metabolism of carcinogens or in the repair of damaged DNA in lung cells, TERT (rs2853669), OGG1 (rs1052133), and CHRNA5 (rs16969968), were examined for association with NSCLC risk in a case-control study of 144 patients and 211 control subjects of Eastern Algerian population in an attempt to explain this interindividual difference.

In our population, none of these three genes shows an influence on the LC risk, except the CHRNA5 rs16969968 SNP, which was significantly different between cases and controls, but after adjustment, the signification disappears. These results are in accordance with the findings of numerous studies but contradictory to others [16, 26, 29]. The allelic frequencies were slightly different from those found in other populations and close to those of Caucasian population but differ drastically from Asiatic and African population [30].

For TERT gene, after sequencing its promoter, we identified in our population the rs2853669 polymorphism. The rs2853669 MAFs in our control group is C = 20%. According to literature and HapMap data, there is no big difference between the MAFs of rs2853669 in Caucasian and Asian populations, C: 26%-37% [12, 31]. Overall, in our study, no significant association was found between the rs2853669 and NSCLC. In the stratified analysis, no difference was noticed for all stratified factors. Only two studies were performed in LC which were conducted exclusively on the Asian population, and they found a strong association between rs2853669 and LC risk [32, 33]. A large recent meta-analysis where thirteen studies involving 16 datasets were pooled to evaluate the association between rs2853669 and cancer risk were realized, and researchers demonstrated that rs2853669 alone does not increase or decrease the overall risk and prognosis of cancer. In a stratified analysis by cancer type, a protective effect was found for breast cancer and a significant association was found for LC and glioblastoma; however, the small number of studies limited its credibility [29]. Concurrently, another recent meta-analysis with the same objective of the precedent had the pooled results indicating that the rs2853669 polymorphism was significantly associated with increased cancer risk in a homozygote model. In the stratified analysis, a significantly increased cancer risk was observed for Asians, but not so for Caucasian patients. A subgroup analysis by cancer type also revealed a significant increase in the risk of LC, but not breast cancer [12].

For the OGG1 rs1052133, previous studies have shown that homozygous carriers of the variant appear to have reduced repair capacity toward oxidized DNA lesions, and previous evidence indicated higher levels of 8-oxoG in lung tissue of LC patients than in lung tissue of patients without cancer [34, 35]. Numerous studies have investigated the association between LC and this SNP, and the results are quite diverse [16–18]. In our study, the MAFs in the control group of rs1052133 was G = 29%, which is slightly higher than frequencies previously reported for Caucasians (15% to 25%), and lower than those reported (40% to 62%) for Asian populations, but consistent with those obtained (33%) for the Turkish population, and similar to those found (G = 27%) in a study of the North African population [16, 36, 37]. The frequency of individuals carrying homozygous variant allele GG was slightly higher in the controls (9.5%) than in the cases (7.6%), which suggests a protective effect, but this difference is not statistically significant (*p* = 0.46). Two previous studies found that this SNP could have a protective effect for LC [36, 38]. Overall, the rs1052133 does not have any effect on the risk of NSCLC in our population. These results are in agreement with the finding of three studies but in discordance with evidence from two other studies [13, 36, 39–41]. Duan et al. did a meta-analysis (data from 8 studies), and no association was found between OGG1 rs1052133 and NSCLC risk [16]. It seems that Asians may have a much higher susceptibility to LC than the others due to having a higher frequency for the variant G allele [42, 43].

In our stratified analysis by histological type, gender, age, and smoking habits, only the age showed a marginally significant elevation of risk of NSCLC for persons more than 60 years old and carrying a recessive genotype GG. However, results obtained by Hung et al. were not in agreement with ours [34].

Our findings could not explain the significantly lower capacity to incise 8-oxoG by leukocytes of NSCLC patients in comparison to healthy controls. A possible explication is that OGG1 gene polymorphisms are only one of many parameters that might affect OGG1 activity. Another explanation as suggested by Lee et al. is that the GG genotype is deficient in the repair of oxidatively generated DNA damage only under conditions of cellular oxidative stress [44]. However, both of these hypotheses need to be confirmed in future studies.

The rs16969968 CHRNA5 polymorphism was the subject of a large number of investigations [21, 23, 26, 45]. Different gene expression and disease association studies provided evidence that both nicotine-dependence risk and LC risk are influenced by this polymorphism. This variant is common in populations of European and Middle Eastern origin, (MAFs = 37%–43%) but uncommon in African, East Asian, and Native American populations (<5%) [20]. For the Algerian population, the MAFs of the rs16969968 in the control group is A = 31%, which is in disagreement with the result found for the Mozabit ethnic group (MAFs = 18%) [46]. This result can be explained because the Mozabit ethnicity stands out genetically from the remaining population due to their practice of endogamy [46]. Our results show a decrease of the variant allele in cases and not in controls, but after adjustment for sex, age, and smoking, the signification disappears (*p* = 0.16). Thus, our data do not support an important role for the SNP in NSCLC risk. However, previous studies on different ethnic groups, predominantly Caucasian, showed a rather important association between rs16969968 and LC risk.

When stratified according to histologic subtype, rs16969968 was associated with LC risk in ADK but not in SCC (*p* = 0.03) under the recessive model. Falvella et al. reported that CHRNA5 mRNA levels are upregulated 30-fold in ADK compared with normal lung tissue in individuals carrying AA genotype compared with those having GG genotype [47]. Nevertheless, Jaworoska et al. showed that this locus is implicated in all histopathologic subtypes of LC [48]. In spite of ADK and SCC being categorized as NSCLC, they have as molecular pattern, and for that reason, the rs16969968 may influence LC susceptibility differently according to histologic subtype. Additional studies are needed to confirm this hypothesis.

The stratified study on the smoking factor showed that smokers with AA genotype exhibit a four-fold higher risk of NSCLC compared with those carrying GA and GG genotypes. Results reported by Le Marchand et al. could explain our results; the authors found that smokers with the AA genotype smoke more cigarettes, but also smoke more intensely, extracting a greater amount of nicotine and carcinogens per cigarette, compared with noncarriers [49]. On the other hand, another plausible explanation is that the variant of rs16969968 leads to reduced receptor activity and that individuals carrying the A allele may require larger amounts of nicotine to achieve the same level of dopamine release [20]. On the other hand, a meta-analysis concluded that the rs16969968-A predicts delayed smoking cessation and an earlier age of LC diagnosis [50]. It was confirmed by a recent study realized by Forget et al. showing that transgenic rats expressing a human nicotinic receptor polymorphism self-administer more nicotine at high doses and exhibit higher nicotine-induced reinstatement of nicotine seeking than wild type. This relapse is associated with reduced neuronal activity in the interpeduncular nucleus [51]. All these studies show the importance of the rs16969968, especially for early diagnosis of LC, and may provide potential targets for new therapies for smoking cessation interventions. Especially, prospective studies indicate that in 2030, tobacco use will be responsible for 10 million deaths, making it the leading cause of preventable deaths [52].

It is important to mention that the Algerian population—which belongs to Africa's largest country—is a very specific population genetically wise due to both historical and cultural reasons with the Berbers being the native population of the region [53]. History of the region bears witness to many invasions, conquests, and migrations by Phoenicians, Romans, Vandals, Byzantines, Arabs, Jews, Spanish, and French, making it a true admixture which could explain why the MAFs of the Algerian population is close to the Caucasian and Middle Eastern ones and that no significant association was found. In addition, it was shown that some Berber populations (Tuareg, Mozabite) are different compared to the genetic North African ones, having gone through long periods of genetic isolation [27]. This can explain the remarkable difference between the rs16969968 CHRNA5 MAFs of Mozabite that were found (A = 18%) and ours (A = 31%) [46]. Another characteristic of our population, related to cultural factors connected with smoking, is that Algerian women do not smoke except in rare cases, which could explain the lack of significance between rs16969968, (strongly associated with the risk of LC) in Caucasian population.

## 5. Conclusions

In conclusion, our study, which is the first of its kind related to the molecular of LC for the East Algerian population, has considered the association of the polymorphisms of three genes TERT (rs2853669), CHRNA5 (rs16969968), and OGG1 (rs1052133) in Eastern Algerian population, and has shown that the rs16969968 CHRNA5 has a significant association with adenocarcinoma LC, and interestingly that the risk is important for smoking patients carrying the AA genotype.

These significant and novel results of our study have however some limitations. First, there is the limited sample size used in the study and the mismatch of our population in sex, age, and smoking, which could result in some bias in the results (although the unconditional logistic regression we used with adjustment for confounding variables, such as sex, age, and smoking, mostly neutralized this effect). Secondly, as the sample was not too large, the statistical power to detect the modest effect of three potential functional SNPs in stratified analyses is relatively low. Besides this, there were some missing information on the smoking status for some people and the duration and intensity of the nicotinic addiction tests. In any case, to fully apprehend the difference in the genetic background of the various North African populations will require more studies, and some of the results reported here may help bring new perspectives on the topic.

## Figures and Tables

**Table 1:** Characteristics of case and control subjects.

Characteristics	Cases (%) (*n* = 144)	Controls (%) (*n* = 211)	*p* value
Sex			<0.01
Male	119 (83)	134 (63.5)	
Female	25 (17)	77 (36.5)	
Age (years)			<0.01
<60	72 (50)	170 (80.5)	
≥60	72 (50)	41 (19.5)	
Mean age (range)	59.85 (26-80)	52.33 (20-88)	
Smoking status			<0.01
Smokers	104 (73)	61 (38)	
Nonsmokers	38 (27)	100 (62)	
No information	2	50	
Histology			/
Adenocarcinoma	96 (66%)	/	
Squamous cell carcinoma	48 (34%)		

*p* value based on Pearson's chi-square test.

**Table 2:** TERT, OGG1, and CHRNA5 genotypes and lung cancer risk.

Genes	Genotype/mode^a^	Cases (%)	Controls (%)	*p*	OR^∗^ (CI)	*p* ^∗^
TERT rs2853669		126	94			
	TT	71 (56.3)	57 (61)	Ref	—	—
	TC	49 (38.9)	36 (38)	0.755	0.99 (0.53–1.89)	0.99
	CC	6 (4,8)	1 (1)	0.11	4.15 (0.61–83.73)	0.21
	T	191 (76)	150 (80)	Ref	—	—
	C	61 (24)	38 (20)		1.22 (0.72–2.07)	0.45
	Recessive			0.12	4.72 (0.68–97.04)	0.17
	Dominant			0.52	1.11 (0.59–2.07)	0.75

CHRNA5^b^ rs16969968		143	211			
	GG	60 (42)	93 (44.3)	Ref	—	—
	GA	64 (44.8)	105 (49.8)	0.8	0.98 (0.58–1.63)	0.93
	AA	19 (13.2)	13 (6.2)	0.03	1.75 (0.68–4.69)	0.25
	G	184 (64.3)	291 (69)	Ref	—	—
	A	102 (35.7)	131 (31)		1.16 (0.80–1.68)	0.41
	Recessive			0.02	1.83 (0.78–4.48)	0.16
	Dominant			0.69	1.07 (0.65–1.76)	0.78

OGG1^b^ rs1052133		144	210			
	CC	79 (54.9)	107 (50.9)	Ref	—	—
	CG	54 (37.5)	83 (39.3)	0.58	0.88 (0.52–1.48)	0.62
	GG	11 (7.6)	20 (9.5)	0.46	1.45 (0.53–4.07)	0.46
	C	212 (73.6)	297 (71)	Ref	—	—
	G	76 (26.4)	123 (29)		1.01 (0.68–1.5)	0.92
	Recessive			0.53	1.44 (0.55–3.83)	0.45
	Dominant			0.46	0.94 (0.57–1.54)	0.79

OR = odds ratio; CI = confidence interval. ^∗^OR adjusted for sex age and tobacco consumption. ^a^The dominant mode: AA vs Aa+aa, where A is major allele and a is minor allele; the recessive mode: AA+Aa vs aa. ^b^Numbers may not add up total number of patients due to genotype failures.

**Table 3:** Association between TERT, CHRNA5, and OGG1 polymorphisms and lung cancer risk, stratified by histology and tobacco consumption.

Population	Gene	Genotype	Cases/controls^a^	*p*	OR_Adj_	CI_Adj_	*P*_Adj_
Smokers^∗^	TERT rs2853669	TT	46/20		Ref		
		TC	40/19	0.81	0.86	(0.39-188)	0.71
		CC	5/0	0.14	—	—	0.99
		Dominant (TC+CC)	46/20	0.93	0.97	(0.44-2.10)	0.94
		Recessive (TC+TT)	86/39	0.13	—	—	0.98
	CHRNA5 **r**s16969968	GG	42/24		Ref		
		GA	44/34	0.37	0.72	(0.36-1.42)	0.35
		AA	18/3	0.05	3.29	(0.79-15.28)	0.08
		Dominant (GA+AA)	62/37	0.89	0.93	(0.48-1.79)	0.83
		Recessive (GA+GG)	86/58	0.02	3.91	(1.24-17.34)	0.03
	OGG1 rs1052133	CC	59/35		Ref		
		CG	36/23	0.82	0.81	(0.40-1.63)	0.56
		GG	9/3	0.40	2.01	(0.53-9.80)	0.32
		Dominant (CG+GG)	45/26	0.93	0.92	(0.48-1.78)	0.82
		Recessive (CG+CC)	95/58	0.37	2.03	(0.57-9.59)	0.30

Nonsmokers^∗^	TERT rs2853669	TT	23/30		Ref		
		TC	9/9	0.62	1.32	(0.42-4.19)	0.63
		CC	1/1	0.85	1.54	(0.05-41.59)	0.76
		Dominant (TC+CC)	10/10	0.61	1.38	(0.46-4.17)	0.55
		Recessive (TC+TT)	32/39	0.89	1.71	(0.06-47.92)	0.71
	CHRNA5 rs16969968	GG	16/49		Ref		
		GA	22/44	0.4	1.36	(0.61-3.05)	0.44
		AA	1/7	0.44	0.33	(0.01-2.49)	0.36
		Dominant (GA+AA)	21/51	0.54	1.19	(0.54-2.63)	0.65
		Recessive (GA+GG)	36/93	0.34	0.31	(0.01-1.97)	0.29
	OGG1 rs1052133	CC	18/48		Ref		
		CG	18/45	0.86	0.94	(0.42-2.10)	0.89
		GG	2/7	0.74	0.83	(0.10 - 4.23)	0.84
		Dominant	20/52	0.94	0.91	(0.42-1.99)	0.82
		Recessive	36/93	0.71	0.84	(0.11-3.80)	0.83

Adenocarcinoma^∗∗^	TERT rs2853669	TT	50/57		Ref		
		TC	33/36	0.88	1.01	(0.50-2)	0.97
		CC	3/1	0.26	3.39	(0.39-72.50)	0.31
		Dominant (TC+CC)	36/37	0.73	1.09	(0.55-2.13)	0.79
		Recessive (TC+TT)	83/93	0.27	3.90	(0.43-85.93)	0.26
	CHRNA5 **r**s16969968	GG	37/93		Ref		
		GA	42/105	0.98	1.01	(0.57-1.79)	0.95
		AA	16/13	0.005	2.54	(0.95-7.07)	0.06
		Dominant (GA+AA)	58/118	0.40	1.18	(0.68-2.06)	0.53
		Recessive (GA+GG)	79/198	0.003	2.55	(1.07-6.35)	0.03
	OGG1 rs1052133	CC	50/107		Ref		
		CG	39/83	0.98	0.95	(0.53-1.67)	0.86
		GG	7/20	0.53	1.61	(0.52-4.80)	0.39
		Dominant (CG+GG)	46/103	0.85	1.00	(0.58-1.73)	0.97
		Recessive (CG+CC)	89/190	0.52	1.49	(0.50-4.24)	0.45

Squamous^∗∗^	TERT rs2853669	TT	21/57		Ref		
		TC	16/36	0.63	1.10	(0.43-2.77)	0.83
		CC	3/1	0.03	10.87	(0.67-573.04)	0.15
		Dominant (TC+CC)	19/37	0.38	1.26	(0.51-3.12)	0.60
		Recessive (TC+TT)	37/93	0.04	7.60	(0.65-208.01)	0.14
	CHRNA5 rs16969968	GG	23/93		Ref		
		GA	22/105	0.61	0.81	(0.37-1.71)	0.58
		AA	3/13	0.91	0.78	(0.13-3.71)	0.76
		Dominant (GA+AA)	25/118	0.62	0.80	(0.38-1.68)	0.56
		Recessive (GA+GG)	45/198	0.98	0.93	(0.18-3.95)	0.93
	OGG1 rs1052133	CC	29/107		Ref		
		CG	15/83	0.24	0.64	(0.28-1.42)	0.28
		GG	4/20	0.60	1.50	(0.31-6.72)	0.59
		Dominant (CG+GG)	19/103	0.23	0.71	(0.33-1.52)	0.39
		Recessive (CG+CC)	44/190	0.79	1.67	(0.38-6.66)	0.46

OR = odds ratio; CI = confidence interval; Adj = adjusted. ^∗^OR adjusted for gender and age. ^∗∗^OR adjusted for gender, age, and smoking habit. ^a^Numbers may not add up the total number of patients due to genotype failures.

**Table 4:** Association between TERT, CHRNA5, and OGG1 polymorphisms and lung cancer risk, stratified by gender and age.

Population	Gene	Genotype	Cases/controls^a^	*p*	OR_Adj_	CI_Adj_	*P*_Adj_
Male^∗∗^	TERT rs2853669	TT	55/40		Ref		
		TC	40/32	0.76	0.74	(0.35-1.55)	0.43
		CC	6/0	0.04	—	—	0.99
		Dominant (TC+CC)	46/32	0.88	0.90	(0.43-1.86)	0.78
		Recessive (TC+TT)	95/72	0.03	—	—	0.98
	CHRNA5 rs16969968	GG	49/60		Ref		
		GA	51/65	0.88	0.92	(0.50–1.71)	0.80
		AA	18/9	0.04	1.99	(0.70–6.26)	0.20
		Dominant (GA+AA)	69/74	0.60	1.05	(0.58–1.90)	0.85
		Recessive (GA+GG)	100/125	0.02	2.09	(0.82–5.88)	0.13
	OGG1 rs1052133	CC	68/67		Ref		
		CG	42/51	0.43	0.80	(0.44-1.48)	0.47
		GG	9/15	0.24	1.49	(0.44-5.52)	0.52
		Dominant (CG+GG)	51/66	0.28	0.87	(0.48-1.56)	0.64
		Recessive (CG+CC)	110/118	0.31	1.51	(0.47-5.36)	0.48

Female^∗∗^	TERT rs2853669	TT	16/17		Ref		
		TC	9/4	0.20	2.49	(0.66-10.91)	0.19
		CC	0/1	0.33	—	—	0.99
		Dominant (TC+CC)	9/5	0.32	2.02	(0.56–7.97)	0.29
		Recessive (TC+TT)	25/21	0.28	—	—	0.99
	CHRNA5 rs16969968	GG	11/33		Ref		
		GA	13/40	0.95	1.03	(0.39-2.70)	0.94
		AA	1/4	0.80	0.75	(0.02-8.22)	0.83
		Dominant (GA+AA)	14/44	0.92	1.01	(0.39-2.62)	0.97
		Recessive (GG+AA)	24/73	0.81	0.85	(0.03-7.46)	0.90
	OGG1 rs1052133	CC	11/40		Ref		
		CG	12/32	0.51	1.06	(0.39-2.85)	0.89
		GG	2/5	0.67	1.23	(0.14–7.25)	0.82
		Dominant	14/37	0.48	1.06	(0.41-2.77)	0.89
		Recessive	23/72	0.79	1.20	(0.16-6.24)	0.83

>60^∗^	TERT rs2853669	TT	32/16		Ref		
		TC	28/5	0.06	2.26	(0.72-7.97)	0.17
		CC	3/0	0.22	—	—	0.99
		Dominant (TC+CC)	31/5	0.04	2.49	(0.80-8.72)	0.12
		Recessive (TC+TT)	60/21	0.30	—	—	0.99
	CHRNA5 rs16969968	GG	31/16		Ref		
		GA	29/23	0.30	0.62	(0.26–1.43)	0.27
		AA	12/2	0.15	2.83	(0.62–20.48)	0.22
		Dominant (GA+AA)	42/93	0.52	1.26	(0.66–2.42)	0.46
		Recessive (GG+AA)	64/159	0.36	1.22	(0.38–3.86)	0.72
	OGG1 rs1052133	CC	38/15		Ref		
		CG	29/26	0.04	0.49	(0.21-1.11)	0.09
		GG	5/0	0.16	—	—	0.99
		Dominant (CG+GG)	34/26	0.09	0.58	(0.25-1.29)	0.18
		Recessive (CG+CC)	67/41	0.08	—	—	0.98

<60^∗^	TERT rs2853669	TT	39/41		Ref		
		TC	21/31	0.34	0.67	(0.30-1.48)	0.33
		CC	3/1	0.30	2.99	(0.33–65.93)	0.37
		Dominant (TC+CC)	24/32	0.49	0.76	(0.34-1.63)	0.48
		Recessive (TC+TT)	60/72	0.24	3.70	(0.41-82.55)	0.28
	CHRNA5 rs16969968	GG	29/77		Ref		
		GA	35/82	0.67	1.26	(0.65–2.44)	0.48
		AA	7/11	0.31	1.27	(0.35–4.48)	0.70
		Dominant (GA+AA)	42/93	0.52	1.26	(0.66–2.42)	0.46
		Recessive (GG+GA)	64/159	0.36	1.22	(0.38–3.86)	0.72
	OGG1 rs1052133	CC	41/92		Ref		
		CG	25/57	0.95	1.33	(0.76–2.66)	0.41
		GG	6/20	0.42	1.01	(0.30–3.20)	0.97
		Dominant (CG+GG)	31/77	0.72	1.27	(0.67–2.44)	0.45
		Recessive (CG+CC)	66/149	0.42	0.90	(0.27–2.75)	0.86

OR = odds ratio; CI = confidence interval; Adj = adjusted. ^∗^OR and *p* adjusted for smoking and gender. ^∗∗^OR adjusted for age and smoking habit. ^a^Numbers may not add up the total number of patients due to genotype failures.

**Table 5:** Association between TERT, CHRNA5, and OGG1 polymorphisms and lung cancer risk, stratified by gender, age, smoking, and histological at the level of alleles.

Population	Gene	Allele	Case/control^a^	*p*	OR_Adj_	CI_Adj_	*P*_Adj_
Smokers^§^	TERT	T	132/59				
		C	50/19	0.60	1.15	0.62–2.18	0.66
	CHRNA5	G	128/82				
		A	80/40	0.30	1.25	0.78–2.03	0.34
	OGG1	C	154/93				
		G	54/29	0.65	1.07	0.64–1.83	0.79

Nonsmokers^§^	TERT	T	55/69				
		C	11/11	0.62	1.36	0.52–3.54	0.52
	CHRNA5	G	52/142				
		A	22/58	0.91	0.98	0.53–1.78	0.95
	OGG1	C	54/141				
		G	22/59	0.93	0.92	0.50–1.67	0.81

Adenocarcinoma^§§^	TERT	T	131/128				
		C	39/30	0.38	1.16	0.66–2.04	0.57
	CHRNA5	G	114/224				
		A	74/98	0.04	1.33	0.89–1.98	0.15
	OGG1	C	137/234				
		G	53/88	0.88	1.07	0.69–1.63	0.75

Squamous^§§^	TERT	T	56/128				
		C	22/30	0.11	1.48	0.71–3.08	0.28
	CHRNA5	G	66/224				
		A	28/98	0.90	0.87	0.49–1.52	0.64
	OGG1	C	71/234				
		G	23/88	0.58	0.88	0.48–1.60	0.70

Male^∗∗^	TERT	T	146/90				
		C	52/24	0.30	1.15	0.64–2.11	0.63
	CHRNA5	G	145/123				
		A	87/57	0.21	1.21	0.78–1.87	0.38
	OGG1	C	175/133				
		G	60/47	0.91	0.98	0.61–1.57	0.93

Female^∗∗^	TERT	T	41/38				
		C	9/6	0.56	1.45	0.47–4.76	0.51
	CHRNA5	G	35/101				
		A	15/41	0.88	0.99	0.47–2.02	0.98
	OGG1	C	34/101				
		G	16/41	0.68	1.07	0.51–2.16	0.85

>60^∗^	TERT	T	92/37				
		C	34/5	0.04	2.21	0.84–6.96	0.13
	CHRNA5	G	91/53				
		A	53/27	0.65	1.12	0.63–2.03	0.69
	OGG1	C	105/55				
		G	39/25	0.51	0.86	0.47–1.61	0.64

<60^∗^	TERT	T	95/91				
		C	27/25	0.91	0.94	0.49–1.79	0.86
	CHRNA5	G	89/171				
		A	49/71	0.21	1.18	0.73–1.90	0.48
	OGG1	C	103/179				
		G	37/63	0.93	1.14	0.68–1.89	0.60

OR = odds ratio; CI = confidence interval; Adj = adjusted. ^∗^OR and *p* adjusted for smoking and gender. ^∗∗^OR adjusted for age and smoking habit. ^a^Numbers may not add up total number of patients due to genotype failures. ^§^OR adjusted for gender and age. ^§§^OR adjusted for gender, age and smoking habit.

## Data Availability

The data used to support the findings of this study are included within the article.
